# Views on the peer review system of biomedical journals: an online survey of academics from high-ranking universities

**DOI:** 10.1186/1471-2288-13-74

**Published:** 2013-06-07

**Authors:** Roger Chun-Man Ho, Kwok-Kei Mak, Ren Tao, Yanxia Lu, Jeffrey R Day, Fang Pan

**Affiliations:** 1 Department of Psychological Medicine, Yong Loo Lin School of Medicine, National University of Singapore, Singapore; 2 Department of Community Medicine and School of Public Health, Faculty of Medicine, University of Hong Kong, Hong Kong; 3 Department of Medicine, Yong Loo Lin School of Medicine, National University of Singapore, Singapore; 4 Faculty of Education, University of Hong Kong, Pokfulam, Hong Kong; 5 Institute of Medical Psychology, School of Medicine, Shandong University, China

**Keywords:** Academics, Peer review, Biomedical journal, Online survey

## Abstract

**Background:**

Peer review is the major method used by biomedical journals for making the decision of publishing an article. This cross-sectional survey assesses views concerning the review system of biomedical journals among academics globally.

**Methods:**

A total of 28,009 biomedical academics from high-ranking universities listed by the 2009 Times Higher Education Quacquarelli Symonds (THE-QS) World University Rankings were contacted by email between March 2010 and August 2010. 1,340 completed an online survey which focused on their academic background, negative experiences and views on biomedical journal peer review and the results were compared among basic scientists, clinicians and clinician scientists.

**Results:**

Fewer than half of the respondents agreed that the peer review systems of biomedical journals were fair (48.4%), scientific (47.5%), or transparent (25.1%). Nevertheless, 58.2% of the respondents agreed that authors should remain anonymous and 64.4% agreed that reviewers should not be disclosed. Most, (67.7%) agreed to the establishment of an appeal system. The proportion of native English-speaking respondents who agreed that the “peer review system is fair” was significantly higher than for non-native respondents (p = 0.02). Similarly, the proportion of clinicians stating that the “peer review system is fair” was significantly higher than that for basic scientists and clinician-scientists (p = 0.004). For females, (β = −0.1, p = 0.03), the frequency of encountering personal attacks in reviewers’ comments (β = −0.1, p = 0.002) and the frequency of imposition of unnecessary references by reviewers (β = −0.06, p = 0.04) were independently and inversely associated with agreement that “the peer review system is fair”.

**Conclusion:**

Academics are divided on the issue of whether the biomedical journal peer review system is fair, scientific and transparent. A majority of academics agreed with the double-blind peer review and to the establishment of an appeal system. Female academics, experience of personal attacks and imposition of unnecessary references by reviewers were related to disagreement about fairness of the peer review system of biomedical journals.

## Background

For centuries, peer review has been the major method to determine the fate of an article submitted to biomedical journals. The process involves inviting reviewers to evaluate an article [1]. They then make recommendations to an editor concerning the decision as to whether or not the article should be published. Fletcher and Fletcher argued that there is no ethical or scientific reason to support that peer review should be the only method to assess articles presented to journals [2]. The current peer review process is based on the fundamental assumption that peer review is objective, rational and free of prejudice [3,4]. Academics have increasingly expressed concern about the review system which seems to result in errors in acceptance or rejection of articles. Examples of acceptance have included misleading reports linking autism and the triple vaccine for measles, mumps and rubella (MMR) [5] and fraudulent cloning work [6]. Another well-known example occurred when a subsequent Nobel laureate’s article on the causes of peptic ulcer was rejected for publication [7]. These instances could be attributed to the bias of editors or reviewers. Journal editors may fail to fulfill their roles and reject articles without a fair review [8] or accept articles as a result of selection bias. On the other hand, reviewers may have bias towards articles due to conflicts of interest (COI) [7], research rivalry, jealousy, or incompetence [8]. Alternatively, confirmatory bias against findings contrary to conventional belief may exist [3]. Wager and Jefferson [9] (2001) highlighted some of the shortcomings of peer review, including its failure to discover errors and fraud, inefficiency, unnecessary delay and potential scientific misconduct when reviewers abuse their power in the peer-review system (e.g. rejecting a paper and plagiarizing its ideas and results) [10].

Anonymous reviewers may also exhibit undesirable behaviors such as unreasonable delay, low quality critiques, personal attacks, abuse of position or breach of confidentiality concerning reviewed articles. The conventional peer review system may not be good enough in scrutinizing the best biomedical science for publication and can be improved [8]. There have been a few initiatives attempting to improve the conventional peer review system, such as removing the anonymity of reviewers [11], publishing reviewers’ comments [8], and providing journal alternatives for articles after rejection [12].

A double-blind review system in which the identities of both authors and reviewers are blind to each other, or a single-blind system where authors are blind to the identities of the reviewers but not *vice versa*, are commonly adopted in biomedical journals. Proponents of an open review system emphasized the restoration of a balance between privilege and responsibility of reviewers. An open system, which identifies the reviewers to the public, may reduce the abuse of anonymity, as well as encouraging reviewers to provide fair and constructive comments, thus enhancing the transparency of any COI amongst the reviewers [13]. Some biomedical academics supported an open peer review system and a double-blind review, which may favour junior researchers [13] and researchers from less reputable institutions [14]. Ultimately, they suggested a system of appeal for authors who feel that their articles have not been fairly reviewed. They also point out that open review has a higher rejection rate compared to closed review [11]. Double-blind review may not be absolutely safe, since removal of the authors’ details from the title page and acknowledgement only guaranteed true anonymity in 50-60% of cases during the review process, particularly in small research fields [15,16]. In addition, language biases may exist in the review process, from submission to acceptance [17]. In many cases native English speakers have advantages over non-native speakers as a result of non-English language bias. The language quality may influence the perception of an article’s scientific quality [4].

Currently, little information exists on the views and experiences of academics concerning the biomedical journal peer review system [18]. The Publishing Research Consortium, which represents a group of publishing societies and publishers published a report in 2008 which showed that journal peer review is widely supported, double-blind review being the most preferred method and long review time being the main cause of dissatisfaction [19]. The feasibility of setting up an appeal system to handle unfairly rejected articles has not been well explored.

The aim of this study was to assess views about the peer review system of biomedical journals among academics from high-ranking universities as determined by the Times Higher Education Quacquarelli Symonds (THE-QS) World University Rankings globally. The primary hypothesis was that most academics would agree that the current peer review system of biomedical journals is fair, transparent and scientific. The secondary hypothesis was that native English-speaking academics would be more likely to agree with the primary hypothesis. In addition, we hypothesized that there would be no significant difference in the agreement with the primary hypothesis between basic scientists, clinician-scientists, and clinicians. Finally, we hypothesized that most academics would support the establishment by journals of an appeal system for article submission. In addition to the above hypotheses, we also examined factors associated with agreement with the primary hypothesis.

## Methods

### Questionnaire development

The authors developed a 28-item anonymous online questionnaire with items under four major themes: 1. Demographic characteristics (age, gender, region of origin, country of current workplace, first language), 2. Academic background (basic scientist, clinician or clinician-scientist, academic discipline, number of scientific papers published in peer review journals and years of experience in conducting biomedical research and publication), 3. Negative experiences with biomedical journal peer review (long review times, personal attacks in reviewers’ comments, breadth of confidentiality of the article and imposition of unnecessary references by reviewers), and 4. Positive aspects of the peer review system of biomedical journals (fairness, scientific credibility, and transparency, conflicts of interest, anonymity and competency of the reviewers, role of the editors, and establishment of an appeal system). In addition, open and qualitative comments on peer review systems were collected. The responses to the items of negative experiences and positive aspects of the current peer review system were constructed as 5-point Likert scales. Higher scores indicate higher frequency of occurrence or stronger agreement.

### Subjects

To better represent the geographical distribution and involvement of scientists and clinicians with adequate publishing experiences in international peer-reviewed journals, the following procedures were implemented. Academics from high-ranking universities were chosen, as these universities have been more heavily involved in research than teaching. The inclusion criteria of universities were 1) being listed in the top 200 universities of the THE – QS World University Rankings (2009), 2) having a school of medicine in the university, 3) the school of medicine having both basic science and clinical departments, 4) the school of medicine having a website which provides email addresses of academic staff. Based on the THE-QS World University Rankings 2009, we then identified a representative sample of universities within different countries across the five continents using a multistage stratification method. At the initial stage, a limit of 5 universities per country and a maximum of 15 countries per continent were set to ensure a representative sample. The universities were first stratified by global ranking and by country, and then further by within-country ranking (i.e. top 5). Where 4 or fewer universities met the criteria from a single country, all of the universities were included. At the final stage of stratification, the universities were chosen according to their countries (a maximum of 15 countries per continent). In case 14 or fewer countries ranked in one continent, all of the universities were included in the study.

### Administration

This was a closed survey and an invitation with a cover letter explaining the purpose of the survey was sent via e-mail to 28,009 biomedical academics from 65 universities worldwide during the period of March to August 2010. A link to the online survey was provided in the email for interested participants to complete the survey. All responses were voluntary and anonymous. There was no advertisement and no incentive for participation. No personal data, such as name of the academics or affiliations were collected in the survey as a measure to protect personal information. Only completed surveys were accepted. All the data were collated via the online questionnaire and stored in a database, which was continuously updated during the survey period. Ethics approval was sought from the ethics committee of School of Medicine, Shandong University and all procedures are in compliance with the Helsinki Declaration.

### Data analysis

The response rate was calculated by dividing the number of forms completed during the study period by the number of invitations sent. Descriptive statistics of the characteristics of respondents and responses to each question are presented. As non-respondents had refused to participate in the survey, no attributable demographic information is available for comparison with respondents. Analyses are based on the respondents’ current working locations (continents), occupations (basic scientist, clinician or clinician-scientist); first languages (English as their first language or English as their second language), with individual data aggregated by calculating proportions per category. Normally distributed data were presented as a mean and 95% confidence interval (95% CI) or standard deviation (SD), whereas data that were not normally distributed were presented as a median and 95% CI. Analysis of Variance (ANOVA) was conducted separately to assess the individual associations of demographics and academic backgrounds, with attitudes towards fairness of the peer review system of biomedical journals. Variables that were found to be significantly associated with the agreement of fairness of the peer review system in the univariate ANOVA (p < 0.05) were included in the final models. We have referred to the Checklist for Reporting Results of Internet E-Surveys (CHERRIES) in our presentations of results [20].

## Results

### Geographical distribution and demographics

**Figure 1:**
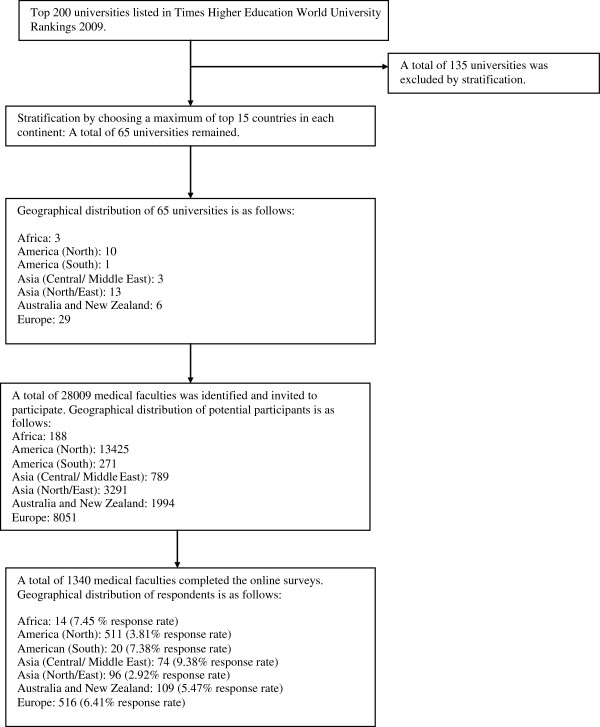
Selection of universities and profile of participants.

We received 1,340 responses from 28,009 invitation sent via emails with an overall response rate of 4.8%. Figure 1 summarizes the selection of universities and response rate per continent. The number of included medical schools included per continent ranged from 1 in South America to 29 in Western Europe; the number of medical schools per country ranged from 1 in Taiwan, New Zealand, Austria, Spain, Italy, Norway, Belgium, Finland, France, Brazil and South Africa; to 5 medical schools in China, Japan, the United States, Canada, Australia, Germany and the United Kingdom. Most universities were in developed countries (84.6%).

Table 1 shows the characteristics of the respondents from 26 different countries. Among the respondents, 70.3% were men and 29.7% were women. Basic scientists made up 560 of the respondents, 516 were clinicians, and 264 were clinician–scientists. The mean age was 52.5 years for men and 48.6 years for women, and 45.6% of respondents reported English as their first language. The median number of scientific papers published in peer review journals was 50 (95% CI: 50–56) and the mean number of years of experience in conducting biomedical research and publication was 20.7 (95% CI: 20.1 - 21.2). The median number of scientific papers published in peer-reviewed journals was similar for clinicians (50, 95% CI: 45–50) and basic scientists (50, 95% CI: 45–60).

**Table 1:** Respondent characteristic by academic background and first language

	**Basic scientists**	**Clinicians**	**Clinician-scientists**	**Chi-squared/t value**	**P value**	**Respondents with English as their first language**	**Respondents with English as their second language**	**Chi-squared/t value/Z value**	**P value**	**All**
	**(n = 560)**	**(n =516)**	**(n = 264)**			**(n = 611)**	**(n = 729)**			**(n = 1340)**
**Gender:**										
Men	354 (63.2%)	380 (73.6%)	208 (78.8%)	25.3	<0.001	409 (66.9%)	533 (73.1%)	6.1	0.01*	942 (70.3%)
Women	206 (36.8%)	136 (26.4%)	56 (21.2%)			202 (33.1%)	196 (26.9%)			398 (29.7%)
**Location**										
Africa	2 (14.3%)	5 (35.7%)	7 (50%)	54.8	<0.001	6 (42.9%)	8 (57.1%)	416.8	<0.001**	14 (1.0%)
America (North)	233 (45.6%)	201 (39.3%)	77 (15.1%)			365 (71.4%)	146 (28.6%)			511 (38.1%)
America (South)	4 (20.0%)	12 (60.0%)	4 (20.0%)			0 (0%)	20 (100%)			20 (1.5%)
Asia (Central/ Middle East)	15 (20.3%)	46 (62.2%)	13 (17.6%)			5 (6.8%)	69 (93.2%)			74 (5.5%)
Asia (North/East)	33 (34.4%)	36 (37.5%)	27 (28.1%)			5 (5.2%)	91 (94.8%)			96 (7.2%)
Australia and New Zealand	52 (47.7%)	42 (38.5%)	15 (13.8%)			95 (87.2%)	14 (12.8%)			109 (8.1%)
Europe	221 (42.8%)	174 (33.7%)	121 (23.4%)			135 (26.2%)	381 (73.8%)			516 (38.5%)
**Mean age (Mean ± SD)**	49.9 ± 11.4	52.6 ± 10.4	52.2 ± 11.2	8.8	<0.001	51.9 ± 11.6	50.9 ± 10.6	1.5	0.1	51.4 ± 11.1
**Median number of papers successfully published in peered- review journals**	50	50	61	9.8	0.007	50	50	0.6	0.5	50
**(Median, 95% CI)**	(45 – 55)	(45 – 60)	(50 – 75)			(50 – 60)	(50 – 59)			(95% CI: 50 – 56)
**Mean number of years of experience in medical research and publications (Mean ± SD)**	20.3 ± 10.6	20.3 ± 10.8	22.4 ± 11.8	3.9	0.02	21.1 ± 11.6	20.4 ± 10.4	1.3	0.2	20.7 ± 11

Data are percentage (number). Sum of percentages equal to 100% in each category. “SD” refers to standard deviation. P values are from t test, χ^2^tests, Mann–Whitney Test and Kruskal-Wallis Test.

Values are numbers and percentages unless stated otherwise.

*p < 0.05; **p < 0.01.

### Positive and negative experiences of journal peer review systems

Table 2 summarizes the perceptions of the biomedical journal peer review system. Among the respondents, less than 50% agreed that biomedical journal peer review is fair (48.4%, 95% CI: 45.8% - 51.1%), transparent (25.1%, 95% CI: 22.8% - 27.5%), and scientific (47.5%, 95% CI: 44.9% - 50.2%). Furthermore, 39.6% (95% CI: 37.0% - 42.3%) agreed that reviewers are competent and 19% (95% CI: 16.9% - 21.1%) agreed that the current system is free from interference. Surprisingly, less than 50% agreed that editors should give every article a fair peer review process, to avoid personal bias (44%, 95% CI: 41.4% - 46.7%) and that editors should screen for unfair comments from reviewers (45.4%, 95% CI: 42.8% - 48.1%). More than 50% of the respondents agreed with the anonymity of authors (58.2%, 95% CI: 55.6% - 60.9%) and reviewers (64.4%, 95% CI: 62.0% - 67.1%). Only 8.6%, (95% CI: 7.1%-10.1%) agreed that reviewers should not be required to declare COI. Most importantly, 67.7% (95% CI: 65.2% - 70.2%) agreed to the need for establishing an appeal system for article submission.

**Table 2:** Undesirable experiences with biomedical journal peer review (all respondents, n = 1,340)

How frequent did each of the following situations happen to the respondents during journal peer review?*	**Very rare/infrequent**	**Sometimes**	**Frequent/all the time**
	**N (%)**	**N (%)**	**N (%)**
The duration of review period is longer than 6 months.	815 (60.8)	374 (27.9)	151 (11.3)
The duration of review is longer than a year.	1231 (91.9)	88 (6.6)	21 (1.6)
Encountering personal attacks in reviewers’ comments.	1078 (80.4)	235 (17.5)	27 (2.0)
Breach of confidentially of articles’ information by reviewers.	1200 (89.6)	126 (9.4)	14 (1.0)
Unauthorised use of articles’ information (e.g. authors’ ideas, data or methods) by reviewers after rejection of articles.	1149 (85.7)	174 (13.0)	17 (1.3)
Imposition of unnecessary references by reviewers.	906 (67.6)	338 (25.2)	96 (7.2)

Items are presented in the order of appearance in the questionnaire.

Percentages sum to 100%.

*Responses on a 5-point Likert Scale: 1, very rare; 2, infrequent; 3, sometimes; 4, frequent; 5, All the time.

Table 3 summarizes the frequencies of undesirable experiences encountered in biomedical journal peer review of all respondents. Among them, 11.3% (95% CI: 9.6% to 13.0%) frequently encountered peer review processes longer than 6 months, and 7.2%, (95% CI: 5.8% to 8.5%) frequently encountered imposition of unnecessary references by reviewers. The other undesirable experiences were relatively less frequent, such as peer attacks in reviewers’ comments (2.0%, 95% CI: 1.3% to 2.8%), review periods longer than a year (1.6%, 95% CI: 0.9% to 2.2%), unauthorised use of an article’s information by reviewers (1.3%, 95% CI: 0.7% to 1.9%), and breach of confidentiality of an article’s information by reviewers (1%, 95% CI: 0.5% to 1.6%).

**Table 3:** Views on biomedical journal peer review (all respondents, n = 1,340)

How much do you agree with the following statements?*	**Strongly disagree/disagree** **n (%)**	**Neutral** **n (%)**	**Strongly agree/agree** **n (%)**
**I) Positive views of the review process**			
Biomedical journal peer review is fair.	304 (22.7)	387 (28.9)	649 (48.4)
Biomedical journal peer review is transparent.	610 (45.5)	393 (29.3)	337 (25.1)
Biomedical journal peer review is scientific.	304 (22.)	399 (29.8)	637 (47.5)
Authors should remain anonymous.	296 (22.1)	264 (19.7)	780 (58.2)
Reviewers should remain anonymous.	271 (20.2)	204 (15.2)	865 (64.4)
Reviewers are competent in general.	306 (22.8)	503 (37.5)	531 (39.6)
**II) Conflict of interest (COI)**			
Reviewers are not required to declare COI.	1125 (84.0)	100 (7.5)	115 (8.6)
The journal review process ensures my article to be free from interference of competitors and people with COI.	659 (49.2)	426 (31.8)	255 (19.0)
**III) Communication**			
After receiving an article, the editors should give every article a fair chance by sending to peer review and avoiding personal bias.	244 (18.2)	506 (37.8)	590 (44.0%)
After receiving reviewers’ feedbacks, editors should screen for unfair reviewers’ comments.	285 (21.3)	446 (33.3)	609 (45.4)
Every biomedical journal should provide an appeal system for authors when their articles are unfairly rejected.	198 (14.8)	235 (17.5)	907 (67.7)

Items are presented in the order of appearance in the questionnaire.

Percentages sum to 100%.

*Responses on a 5-point Likert Scale: 1, very rare; 2, infrequent; 3, sometimes; 4, frequent; 5, All the time.

### Comparisons of responses between native and non-native English-speaking respondents

Table 4 compares undesirable experiences and views on biomedical journal peer review between native and non-native English-speaking respondents. For undesirable experiences, the proportion of non-native English-speaking respondents reporting frequent occurrence of personal attacks (χ^2^ = 11.0, p = 0.004) and imposition of unnecessary references (χ^2^ = 363.4, p = 0.01) was significantly higher than that among native English-speaking respondents.

**Table 4:** Comparison of responses between native and non-native English speaking respondents

	**English as the first language**			**English as the second language**				
	**Rare/infrequent**	**Sometimes**	**Frequent/all the time**	**Rare/infrequent**	**Sometimes**	**Frequent/all the time**	**Chi squared value**	**p value**
	**N (%)**	**N (%)**	**N (%)**	**N (%)**	**N (%)**	**N (%)**		
**Undesirable experiences**								
Personal attacks	512 (83.8)	93 (15.2)	6 (1.0)	566 (77.7)	142 (19.5)	21 (2.9)		
Breach of confidentiality	552 (90.4)	55 (9.0)	4 (0.7)	648 (88.8)	71 (9.7)	10 (1.3)		
Unauthorised use of articles’ information after rejection of articles	528 (86.4)	75 (12.3)	8 (1.3)	621 (85.2)	99 (13.6)	9 (1.2)		
Imposition of unnecessary references	435 (71.2)	130 (21.3)	46 (7.5)	471 (64.6)	208 (28.5)	50 (6.8)		
	**Strongly disagree/Disagree**	**Neutral**	**Strongly agree/Agree**	**Strongly disagree/Disagree**	**Neutral**	**Strongly agree/Agree**	**Chi squared value**	**p value**
	**N (%)**	**N (%)**	**N (%)**	**N (%)**	**N (%)**	**N (%)**		
**Journal peer review is:**								
fair	122 (19.9)	169 (27.7)	320 (52.3)	182 (25.0)	218 (29.9)	329 (45.2)	7.8	0.02*
transparent	276 (45.2)	172 (28.2)	163 (26.7)	334 (45.8)	221 (30.3)	174 (23.8)	1.6	0.5
scientific	158 (25.8)	184 (30.1)	269 (44.0)	146 (20.0)	215 (29.5)	368 (50.5)	7.9	0.02*
free from interference	299 (48.9)	184 (30.1)	128 (21.0)	360 (49.4)	242 (33.2)	127 (17.5)	3.2	0.2
**Editors should:**								
give an article a fair hearing by sending to peer review and avoid personal bias	109 (17.9)	207 (33.9)	295 (48.3)	135 (18.5)	299 (41.0)	295 (40.5)	9.2	0.01*
screen for unfair comments	113 (18.5)	196 (32.1)	302 (49.4)	172 (23.6)	250 (34.3)	307 (42.1)	8.5	0.01*
**Reviewers**								
Anonymity of reviewers	125 (20.5)	85 (13.9)	401 (65.6)	146 (20.0)	119 (16.3)	464 (63.7)	0.5	0.5
Not require to declare conflict of interest	552 (90.4)	25 (4.1)	34 (5.6)	573 (78.6)	75 (10.3)	81 (11.1)	34.5	<0.001**
Competent in general	153 (25.1)	206 (33.7)	252 (41.2)	153 (21.0)	297 (40.7)	279 (38.3)	7.5	0.02*
**Anonymity of authors**	144 (23.6)	119 (19.5)	348 (56.9)	152 (20.8)	145 (19.9)	432 (59.3)	1.4	0.5
**Appeal system in every journal**	98 (16.0)	90 (14.7)	423 (69.3)	100 (13.7)	145 (19.9)	484 (66.4)	6.7	0.04*

*p < 0.05; **p < 0.01.

For the views on biomedical journal peer review, the proportion of native English-speaking respondents who agreed that peer review is fair (χ^2^ = 7.8, p = 0.02) and to the establishment of an appeal system (χ^2^ = 6.7, p = 0.04) was significantly higher than for non-native counterparts. The proportion of native English-speaking respondents who disagreed that reviewers should be exempted from declaring COI (χ^2^ = 34.5, p < 0.001), editors should give an article a fair hearing and be free from personal bias (χ^2^ = 9.2, p = 0.01) and they should screen for unfair comments (χ^2^ = 8.5, p = 0.01) was significantly higher than for non-native English speakers. In contrast, the proportion of non-native English-speaking respondents who agreed that journal peer review is scientific (χ^2^ = 7.9, p = 0.02) and remained neutral about the competency of reviewers (χ^2^ = 7.5, p = 0.02) was significantly higher than for native English-speaking respondents.

### Comparison of responses among basic scientists, clinicians and clinician-scientists

Table 5 shows the comparisons of responses among basic scientists, clinicians and clinician-scientists. The association between the frequencies of undesirable experiences and types of academics was significant. The proportion of clinicians who reported rare or infrequent occurrences of personal attacks by reviewers (χ^2^ = 19.7, p = 0.001), breach of confidentiality by reviewers (χ^2^ = 25.7, p < 0.001), unauthorised use of an article’s information by reviewers after rejection of the article (χ^2^ = 19.5, p = 0.001), and imposition of unnecessary references by reviewers (χ^2^ = 26.7, p < 0.001) was significantly higher than for basic scientists and clinician-scientists.

**Table 5:** Comparison of responses among basic scientists, clinicians and clinician-scientists

	**Basic scientists**			**Clinicians**			**Clinicians scientists**			
	**Rare /infrequent**	**Sometimes**	**Frequent/all the time**	**Rare/infrequent**	**Sometimes**	**Frequent/all the time**	**Rare/infrequent**	**Sometimes**	**Frequent/all the time**	**p value**
	**N (%)**	**N (%)**	**N (%)**	**N (%)**	**N (%)**	**N (%)**	**N (%)**	**N (%)**	**N (%)**	
**Undesirable experiences**										
**Personal attacks**	442 (78.9)	100 (17.9)	18 (3.2)	439 (85.1)	74 (14.3)	3 (0.6)	197 (74.6)	61 (23.1)	6 (2.3)	0.001**
**Breach of confidentiality**	499 (89.1)	56 (10.0)	5 (0.9)	484 (93.8)	28 (5.4)	4 (0.8)	217 (82.2)	42 (15.9)	5 (1.9)	<0.001**
**Unauthorised use of articles’ information after rejection of articles**	485 (86.6)	71 (12.7)	4 (0.7)	458 (88.8)	50 (9.7)	8 (1.6)	206 (78.0)	53 (20.1)	5 (1.9)	0.001**
**Imposition of unnecessary references**	363 (64.8)	154 (27.5)	43 (7.7)	389 (74.5)	99 (19.2)	28 (5.4)	154 (58.3)	85 (32.2)	25 (9.5)	<0.001**
	**Strongly disagree/Disagree**	**Neutral**	**Strongly agree/Agree**	**Strongly disagree/Disagree**	**Neutral**	**Strongly agree/Agree**	**Strongly disagree/Disagree**	**Neutral**	**Strongly agree/Agree**	**p value**
	**N (%)**	**N (%)**	**N (%)**	**N (%)**	**N (%)**	**N (%)**	**N (%)**	**N (%)**	**N (%)**	
**Journal peer review is:**										
**fair**	137 (24.5)	166 (29.6)	257 (45.9)	97 (18.1)	137 (26.6)	282 (54.7)	70 (26.5)	84 (31.8)	110 (41.7)	0.004**
**transparent**	250 (44.6)	176 (31.4)	134 (23.9)	225 (43.6)	152 (29.5)	139 (26.9)	135 (51.1)	65 (24.6)	64 (24.2)	0.2
**scientific**	117 (20.9)	167 (29.8)	276 (49.3)	119 (23.1)	152 (29.5)	245 (47.5)	68 (25.8)	80 (30.3)	116 (43.9)	0.6
**free from interference**	283 (50.5)	179 (32.0	98 (17.5)	228 (44.2)	167 (32.4)	121 (23.4)	148 (56.1)	80 (30.3)	36 (13.6)	0.004**
**Anonymity of authors**	138 (24.6)	115 (20.5)	307 (54.8)	89 (17.2)	99 (19.2)	328 (63.6)	69 (26.1)	50 (18.9)	145 (54.9)	0.009**
**Appeal system in every journal**	93 (16.6)	100 (17.9)	367 (65.5)	74 (14.3)	95 (18.4)	347 (67.2)	31 (11.7)	40 (15.2)	193 (73.1)	0.2

*p < 0.05; **p < 0.01.

As for views on biomedical journal peer review, the proportion of clinicians who agreed that journal peer review is fair (χ^2^ = 15.2, p = 0.004) and to the anonymity of authors (χ^2^ = 13.6, p = 0.009) was significantly higher than for basic scientists and clinician-scientists. The proportion of clinician-scientists who agreed that journal peer review is free from interference (χ^2^ = 15.6, p = 0.004) was significantly lower than for basic scientists and clinicians.

**Figure 2:**
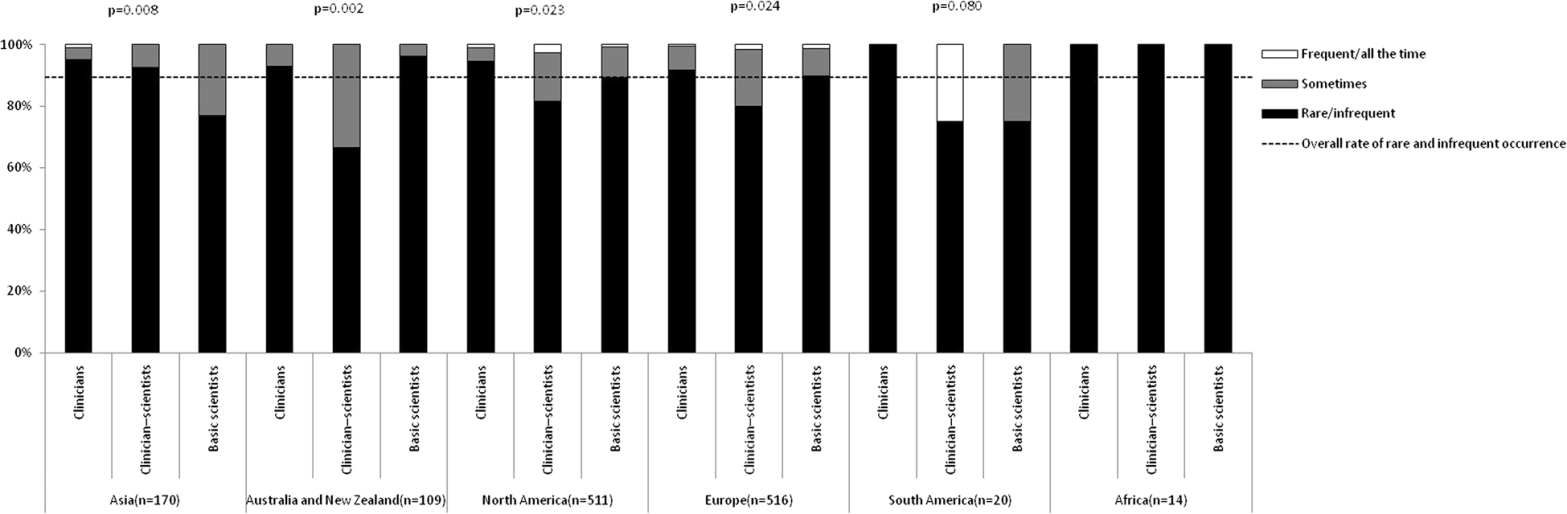
Proportion of frequencies of breach of confidentiality by reviewers encountered by respondents, according to academic background and continent.

**Figure 3:**
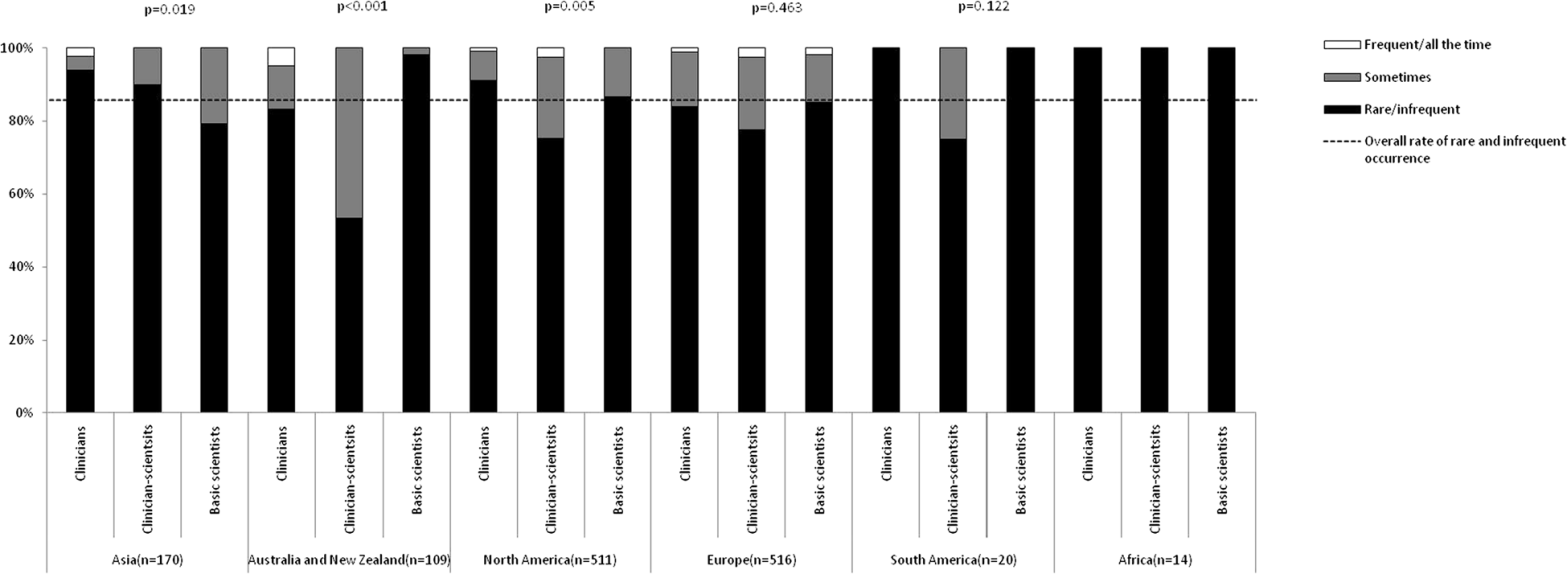
Proportion of frequencies of unauthorized use of articles’ information (e.g. authors’ idea, data or research methods) by reviewers after rejection of articles, according to academic background and continent.

**Figure 4:**
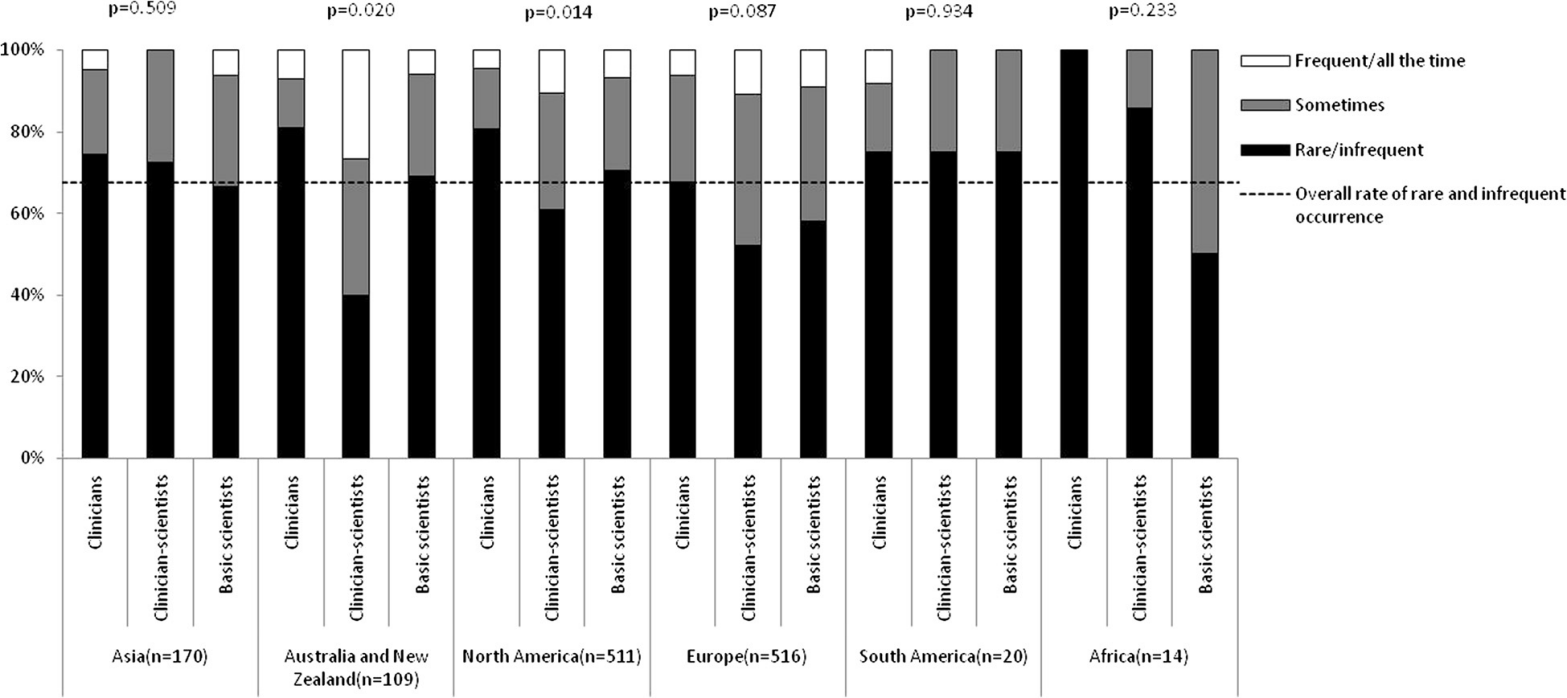
Proportion of frequencies of imposition by reviewers to include unnecessary references according to academic background and continent.

Figure 2 shows the frequencies of breach of confidentiality reported by respondents according to academic background and continent. Among the entire sample, 89.6% (95% CI: 87.9% to 91.2%) reported rare or infrequent occurrences of breach of confidentiality. The proportion of frequently encountering breach of confidentiality was significantly different among clinicians, clinician-scientists and scientists in Asia (χ^2^ = 13.8, p = 0.008; significantly more frequent among basic scientists), Australia and New Zealand (χ^2^ = 12.5, p = 0.002; significantly more frequent among clinician-scientists), North America (χ^2^ = 11.4, p = 0.023; significantly more frequent among clinician-scientists) and Europe (χ^2^ = 11.2, p = 0.024; significantly more frequent among clinician-scientists). No significant difference was found in South America and Africa. For the frequencies of unauthorised use of articles’ information by reviewers after rejection of articles, 85.7% (95% CI: 83.9% to 87.6%) reported rare or infrequent occurrences (see Figure 3). The proportion of frequencies was significantly different among the three types of biomedical academic in Asia (χ^2^ = 11.8, p = 0.019; significantly more frequent among basic scientists), Australia and New Zealand (χ^2^ = 25.5, p < 0.001; significantly more frequent among clinician-scientists), North America (χ^2^ = 15.8, p = 0.005; significantly more frequent among clinician-scientists), but no significant difference was found in Europe, South America and Africa. For the frequencies of imposition of unnecessary references by reviewers, 67.6% (95% CI: 65.1% to 70.1%) reported rare or infrequent occurrences (see Figure 4). The proportion of frequencies of imposition of unnecessary references by reviewers was significantly different among three types of biomedical academics in Australia and New Zealand (χ^2^ = 11.6, p = 0.02, significantly more frequent among clinician-scientists) and North America (χ^2^ = 12.5, p = 0.014, significantly more frequent among clinician-scientists) but not in Asia, Europe, South America or Africa.

### Comparison of responses among different specialties

**Figure 5:**
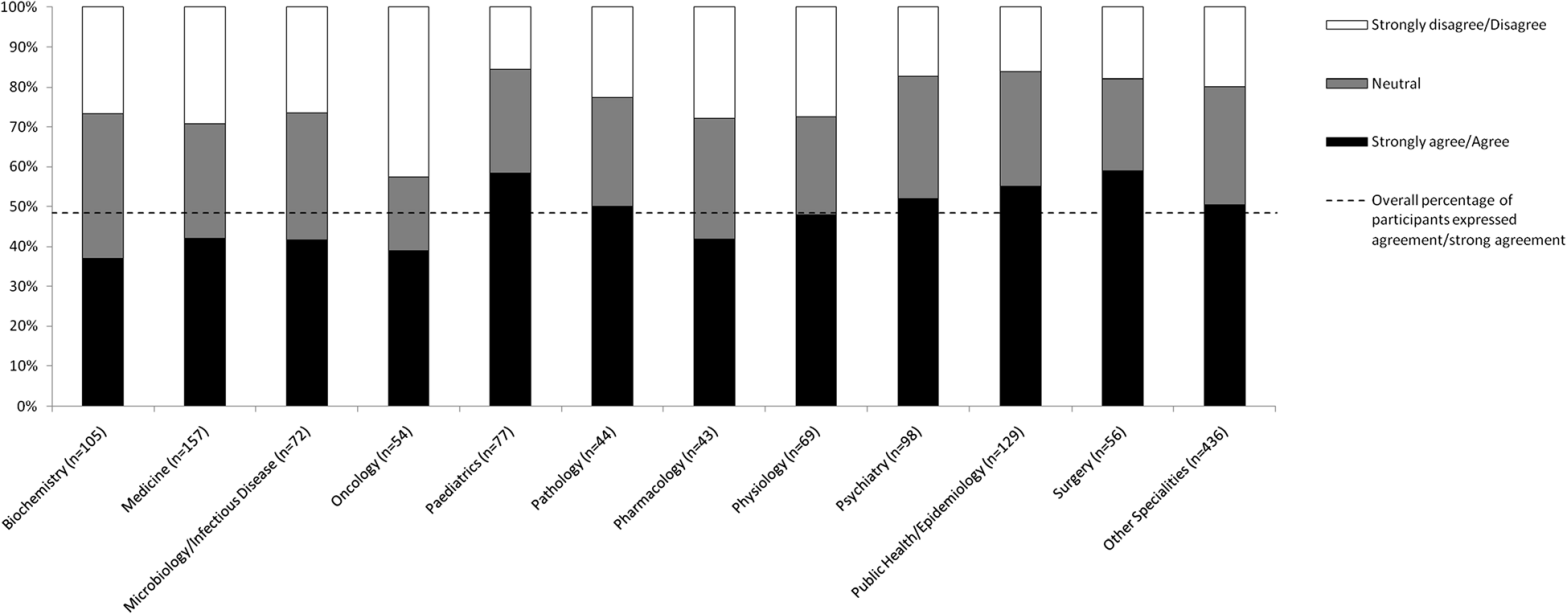
Proportion of agreement with peer review being fair by respondents, according to specialty.

**Figure 6:**
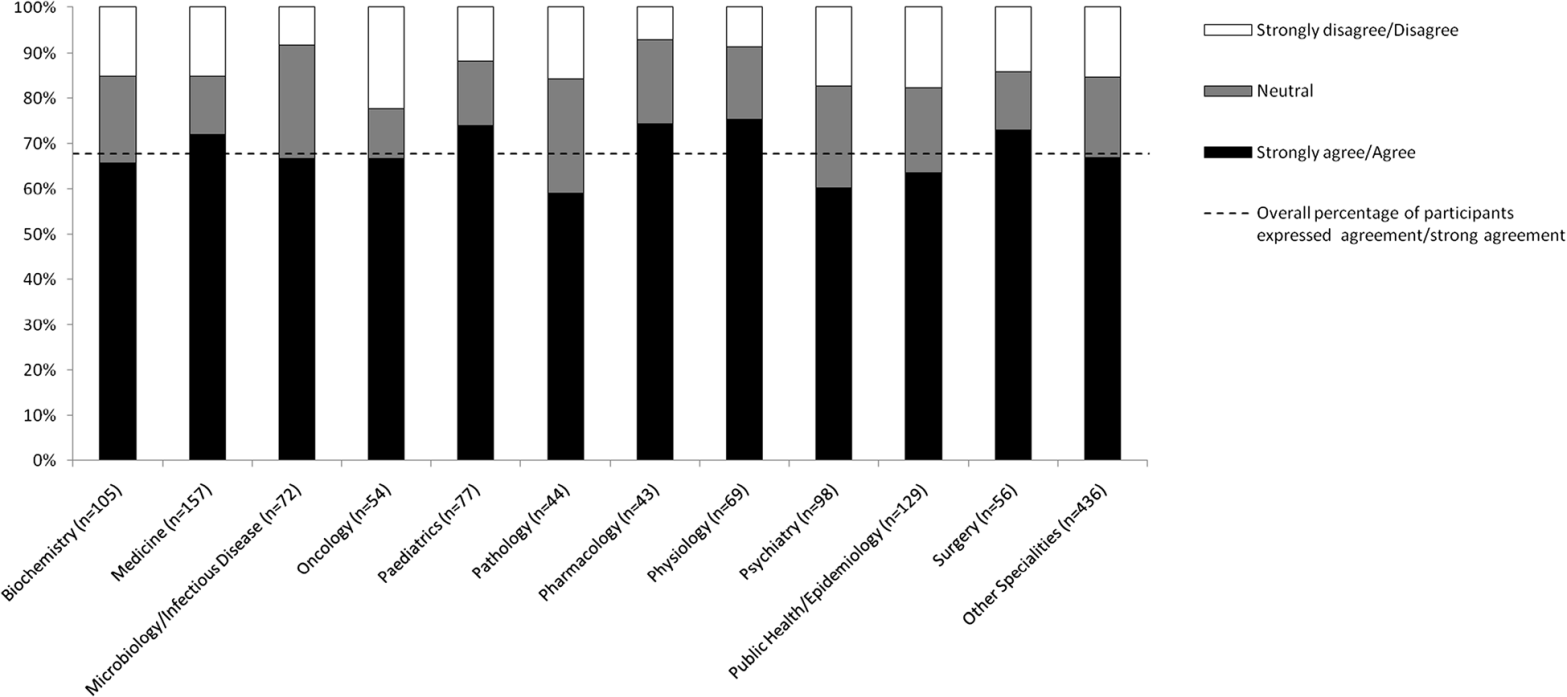
Proportion of agreement to the establishment of an appeal system within the journal by respondents, according to specialty.

Figures 5 and 6 show the proportion of agreement with biomedical journal peer review being fair and wishing to establish an appeal system in journals by respondents, according to their speciality. Among the entire sample, 48.4% (95% CI: 45.8% to 51.1%) agreed that biomedical journal peer review is fair (see Figure 5). Surgery had the highest proportion of respondents expressing agreement and strong agreement (58.9%, 95% CI: 46.0% to 71.8%), followed by Paediatrics (58.4%, 95% CI: 47.4% to 69.5%) and Public Health/Epidemiology (55.0%, 95%CI: 46.5% to 63.6%). Similarly, 67.7% (95% CI: 65.2% to 70.2%) wished for the establishment of an appeal system within the journal for handling unfairly rejected articles (see Figure 6). Across the disciplines, Physiology (75.4%, 95% CI: 65.2% to 85.5%), followed by Pharmacology (74.4%, 95% CI: 61.4% to 87.5%), Pediatrics (74.0%, 95% CI: 64.2% to 83.8%), and Surgery (73.2%, 95% CI: 61.6% to 84.8%) had the highest proportion of respondents expressing agreement or strong agreement with this proposal.

### Factors associated with agreement that biomedical journal peer review is fair

We undertook a univariate ordinal regression analysis, considering the degree of agreement with fairness in journal peer review as the ordinal dependent variable while considering demographics, academic background, personal experiences and views to peer review as independent (explanatory) variables as listed in Table 6. Female gender (β = −0.1, p = 0.03), frequency of encountering personal attacks in reviewers’ comments (β = −0.1, p = 0.002) and frequency of imposition of unnecessary references by reviewers (β = −0.06, p = 0.04) were independently and inversely associated with agreement that biomedical journal peer review is fair. We further performed multivariate ordinal regression using the three variables (female gender, frequency of encountering personal attacks in reviewers’ comments and frequency of imposition of unnecessary references by reviewers) as covariates but could not find a suitable multivariate regression model.

**Table 6:** Univariate regression analysis of the association between degree of agreement that biomedical journal peer review is fair and demographics, academic background and frequency of undesirable experiences encountered

	**Regression coefficient**	**Standard error**	**F change**	**P**
**Demographics**				
Age	−0.001	0.003	0.1	0.7
Gender (Female)	−0.1	0.05	4.9	0.03*
English as first language	−0.05	0.06	0.7	0.4
**Academic background**				
Number of publications	<0.001	<0.001	0.2	0.7
Years of experience in biomedical research	0.001	0.003	0.07	0.8
**Frequency of undesirable experiences encountered (Yes or No)**				
Review times longer than one year	−0.03	0.04	0.9	0.4
Personal attacks in reviewers’ comments	−0.1	0.03	9.9	0.002**
Breach of confidentiality by reviewers	−0.022	0.04	0.4	0.6
Unauthorised use of articles’ information after rejection of articles	−0.022	0.03	0.4	0.5
Imposition of unnecessary references by reviewers	−0.06	0.03	4.4	0.04*

*p < 0.05; **p < 0.01.

#### Open and qualitative comments from respondents

We received comments from 493 respondents (36.8%). We could classify 168 comments into the following common themes: authors should remain anonymous (n = 47); peer review should be transparent and open (n = 41); the current journal peer review is supported (n = 21); reviewers should be rewarded or receive financial enticements (n = 20); reviewers should be experienced and professional (n = 13); journal peer review should be more efficient (n = 12); there is no better option than the current journal peer review system (n = 10) and training should be required for reviewers (n = 4).

## Discussion

Journal peer review is a process in which the work of an academic is subject to an examination by other experts in the same field [21]. Although the first peer review in biomedical journals dates back to 1731 with the publication of “Medical Essays and Observations” by the Royal Society of Edinburgh [14], the process itself and perception from biomedical academics has scarcely been studied for the past three centuries. In this study, 1,340 academics working for high-ranking universities from 26 countries shared their views on peer review in biomedical publication. The results of this survey do not support our primary hypothesis that most biomedical academics would agree that journal peer review is fair, transparent and scientific. Our survey reports that slightly less than half of respondents agreed that the process is fair and scientific, and only about one-quarter agreed that peer review is transparent. Our findings also suggest that peer review, although being the most prevalent system adopted by biomedical journals to select articles for publication, is perceived differently by academics regarding its fairness, scientific credibility and transparency. Our findings correspond to concerns raised by other researchers that there is a need to improve clarity and transparency of biomedical journal peer review [22]. This is perhaps not surprising as peer review depends on unquantifiable perceptions [22]. Decisions on acceptance or rejection of a article should be based on objective selection criteria (e.g. quality scores for an article) and broad criteria (e.g. importance, usefulness, relevance, methods, ethics and accuracy) [22]. Such criteria should be made transparent to authors, editors, reviewers and readers [23]. Measures such as publishing reviewers’ reports, authors’ responses and editors’ comments would increase the transparency of the peer review process [24]. Although some journals have introduced an open peer-review process whereby the identity of authors and peer reviewers are published with the intention to improve transparency [13,25], our respondents generally did not support an open peer-review process as about two-third of respondents agreed that reviewers should remain anonymous. Similarly, about two-thirds of respondents agreed that authors should remain anonymous to reviewers. Double anonymity may provide a safe place for reviewers and authors to exchange frank and sensitive comments [12]. Furthermore, this may have value in concealing the gender of the authors as female gender was a significant factor associated with perception of unfairness in journal peer review. Our findings correspond with the report published by the Publishing Research Consortium (2008) which states that double-blind review was preferred and seen as most effective [19] although single-blind review is more common in biomedical journals.

The proportion of native English-speaking respondents agreeing that journal peer review is fair was significantly higher than for non-native respondents. It is not surprising because the frequencies of non-native English-speaking respondents encountering personal attacks and imposition of unnecessary references were higher compared to native respondents. Nevertheless, there was no significant difference between the two groups on their views of transparency. Interestingly, the proportion of non-native English speaking respondents who agreed that journal peer review is scientific was significantly higher than for native respondents. This result may reflect a higher expectation about journal peer review adopted by native English-speaking biomedical academics. This interpretation is further supported by the results of this survey, as there was a significantly higher proportion of native English-speaking respondents who urged the editors to give an article a fair hearing without personal bias and to screen for unfair comments, for the reviewers to declare COI, as well as for the journals to establish an appeal system.

Our survey classified the respondents according to type of academic and medical speciality. The proportion of clinicians who agreed that biomedical journal peer review is fair was significantly higher than for basic scientists and clinician-scientists, thus contradicting our hypothesis. A possible explanation for this result is that the proportion of basic scientists and clinician-scientists who reported the occurrence of peer review misconduct such as personal attacks and imposition of unnecessary references was significantly higher than clinicians. Academics involving in basic science research may need more safeguards from journal peer review misconduct when publishing their results. The result concerning our final hypothesis, that most academics would support the establishment of an appeal system within the journal to handle unfairly rejected articles, is supported. About 7 in 10 respondents agreed to the establishment of an appeal system.

The results of this study may generate further discussion to improve biomedical journal peer review. The roles of editors involve assessing the external validity of the article with considerations of the appropriateness of the research to the readership of the journal and appointing experienced experts from the same field to review articles [21]. It is interesting to note that about one-third of respondents provided a neutral view on whether editors should give an article a fair hearing and avoid personal bias (37.8%) and screen for unfair reviewers’ comments (33.3%). The proportion of subjects remaining neutral on the editor’s role is almost twice that remaining neutral on establishing an appeal system (17.5%). These findings suggest that the respondents do not have a high expectation concerning editors, because editors are mostly perceived as sending articles out for peer review [9].

The roles of reviewers include assessing the internal validity of an article, such as accuracy and correctness with considerations of the study design, research methodology, statistical analysis and interpretation of results. Reviewers are also expected to provide constructive feedback to enhance scientific quality of the articles and stimulate further consideration by the authors [21]. Findings from our survey confirm that misconduct such as breach of confidentiality and unauthorised uses of article information among reviewers was relatively uncommon. Nevertheless, frequency of encountering personal attacks in reviewers’ comments and frequency of imposition of unnecessary references by reviewers were significant factors associated with perception of unfairness in journal peer review. These findings highlight the importance of publication ethics guidelines which should define and prohibit personal attacks by reviewers. Furthermore, editors should investigate such incidents. A structured and standardized training course for reviewers concerning proper criteria for prompt reviewing may avoid some the pitfalls [14] and enhance the quality of articles. While it is difficult to define unnecessary references, reviewers should try to avoid imposing upon authors to quote the reviewers own publications as it could lead to potential COI. The COI given by reviewers need to be taken seriously in biomedical publication due particularly to the influence of pharmaceutical companies. Not surprisingly, about 8 in 10 respondents disagreed that the reviewers should be exempted from declaring COI. It is interesting to note that a small number of respondents suggested the offering of rewards or financial incentives to reviewers and the report from Peer Review Consortium (2008) also provided limited support for this proposal [19]. Caution is needed in reacting to this suggestion. Firstly, it could increase the production costs of the journal and the costs would be transferred to the subscribers. Secondly, it may lengthen the review times and increase rejection rates in journals which do not offer financial incentives.

To the best of our knowledge, this study is one of the few examining the views on and experiences with biomedical journal peer review among academics. The study has collected important information which identifies directions for improvement of journal review systems. Nevertheless, our study has some limitations. First, it was not based on random selection of medical schools and academics, it mainly focused on the respondents from high-ranking universities with extensive publication experience. Our study may lead to selection bias because we could not identify academics working in low-ranking universities who may encounter more difficulty in publication and it is difficult to set criteria to define ‘low ranking’. As a result, our findings cannot be generalized to academics with relatively less publication experience, academics working in medical schools which only focus on teaching and academics working in a research institute not affiliated with a university. Second, the medical schools were selected based on the THE-QS World University Rankings which have been criticized for their construct validity and possible selection biases of peer reviewers [26]. Third, the present survey method may have under-represented those respondents from Africa, South America and southern Asia. Biomedical research in Mainland China has increased substantially in the 21st Century [27]. Fourth, the response rate (4.8%) was slightly lower than the study conducted by the Publishing Research Consortium (7.7%) in 2008. The low response rate could be due to the fact that academics from high ranking universities are usually successful in getting their manuscripts published and they are less inclined to participate in our survey to express their views. Fifth, the online questionnaire was not able to explore further experiences encountered by participants and focus group interviews would be useful. Furthermore, we did not assess respondents’ views on new review methods such as post-publication peer review and multi-stage open peer review. Jefferson et al. (2002) proposed a randomized controlled study to compare the outcomes of articles undergoing the conventional peer review and an alternative type of assessments [27]. The multi-stage open peer review is a possible alternative method which should be studied further. The multi-stage open peer review involves three stages [28]. The first involves rapid pre-screening and publication as a discussion paper in an online forum. The second involves an 8-week interactive public discussion between the authors and the scientific community. The discussion would be published online and the names of referees may be published. The third stage involves the traditional peer review. The multi-stage open peer review may further enhance the transparency of peer review and the quality of articles as compared to the conventional method.

## Conclusions

We conclude that academics are divided on the issue of whether biomedical journal peer review is fair, scientific and transparent. Undesirable experiences such as personal attacks and breach of confidentiality by reviewers are infrequent. Native English-speaking academics are more likely to agree that biomedical journal peer review is fair when compared with non-native speaking academics. Similarly, clinicians are more likely to agree that journal peer review is fair when compared with basic scientists and clinician-scientists. Our findings suggest that a majority of biomedical academics support anonymity of both authors and reviewers, and the establishment of an appeal system within the journal to handle unfairly rejected articles. Establishing an appeal system or a channel for authors to express concern may be a good step forward in devising strategies to promote fairness in biomedical journal peer review. Further study is required to assess journal editors’ view of developing an appeal system. The female gender, frequency of encountering personal attacks in reviewers’ comments and frequency of imposition of unnecessary references by reviewers are the most significant factors associated with disagreement that biomedical journal peer review is fair. Biomedical journals may consider issuing publication ethics guidelines, offering courses for reviewers, providing authors with channels to expressing their concerns and the adoption of multi-stage open peer review.

## Competing interests

The authors declare that they have no competing interest.

## Authors’ contributions

RCH drafted and revised the article and designed the statistical analysis. KKM revised the article with scientific insight. RT sent out the email invitation and prepared the dataset for analysis. YXL revised the article and references. JRD revised the article and improved the language. FP sought ethics approval and revised the article. All authors read and approved the final manuscript.

## Pre-publication history

The pre-publication history for this paper can be accessed here:

http://www.biomedcentral.com/1471-2288/13/74/prepub
